# Locally indistinguishable orthogonal product bases in arbitrary bipartite quantum system

**DOI:** 10.1038/srep31048

**Published:** 2016-08-09

**Authors:** Guang-Bao Xu, Ying-Hui Yang, Qiao-Yan Wen, Su-Juan Qin, Fei Gao

**Affiliations:** 1 State Key Laboratory of Networking and Switching Technology, Beijing University of Posts and Telecommunications, Beijing, 100876, China; 2 College of Mathematics and Systems Science, Shandong University of Science and Technology, Qingdao, 266590, China; 3 School of Mathematics and Information Science, Henan Polytechnic University, Jiaozuo, 454000, China

## Abstract

As we know, unextendible product basis (UPB) is an incomplete basis whose members cannot be perfectly distinguished by local operations and classical communication. However, very little is known about those incomplete and locally indistinguishable product bases that are not UPBs. In this paper, we first construct a series of orthogonal product bases that are completable but not locally distinguishable in a general *m* ⊗ *n* (*m* ≥ 3 and *n* ≥ 3) quantum system. In particular, we give so far the smallest number of locally indistinguishable states of a completable orthogonal product basis in arbitrary quantum systems. Furthermore, we construct a series of small and locally indistinguishable orthogonal product bases in *m* ⊗ *n* (*m* ≥ 3 and *n* ≥ 3). All the results lead to a better understanding of the structures of locally indistinguishable product bases in arbitrary bipartite quantum system.

The following situation is often encountered in quantum cryptography^1^ and quantum algorithms^2^. Suppose that Alice and Bob share a bipartite quantum system. They are told that the state they own comes from a set of orthogonal states that are known to each of them, but they are not told that which state their combined system is in. What they need to do is to identify the given state by local operations and classical communication (LOCC) since they are in two different places. It is well known that orthogonal quantum states can always be distinguished by global operations. However, this is not always true if we restrict the set of actions on the bipartite system to LOCC only. Bennett *et al.*^3^ first constructed a set of nine orthogonal product states that cannot be perfectly distinguished by LOCC in a 3 ⊗ 3 quantum system. Their work showed the counterintuitive phenomenon of nonlocality without entanglement, i.e., entanglement is not necessary for the local indistinguishability of orthogonal quantum states. Later, a simple proof for the nonlocality of the nine product states was given by Walgate *et al.*^4^. Inspired by their work, many scholars are engaged in the research of the local distinguishability of orthogonal product states. With further research, numerous results^5^,^6^,^7^,^8^,^9^,^10^,^11^,^12^,^13^,^14^,^15^,^16^,^17^,^18^,^19^,^20^ have been presented up to now.

Unextendible product basis (UPB) is a set of orthogonal product states that spans a subspace whose complementary subspace contains no product state. As an object with rich mathematical structure, it was introduced by Bennett *et al.*^21^ and has been thoroughly studied in the literatures^6^,^7^,^8^,^17^,^20^. Bennett *et al.* presented two different UPBs, each of which has five product states in a 3 ⊗ 3 quantum system. Furthermore, they proved that a UPB cannot be perfectly distinguished by LOCC. DiVincenzo *et al.*^20^ gave a complete characterization of unextendible product bases by orthogonality graphs and presented several generalizations of UPBs to arbitrary high dimensions and multipartite systems. Chen *et al.*^6^ made a further research on the minimum size of unextendible product bases. On the other hand, many huge advances have been made on the orthogonal product states that cannot form a UPB. Yu *et al.*^22^ constructed 2*d* − 1 orthogonal states that are locally indistinguishable in *d* ⊗ *d* (*d* ≥ 3) and conjectured that any set of no more than 2(*d* − 1) product states is locally distinguishable in a *d* ⊗ *d* (*d* ≥ 3) quantum system. Wang *et al.*^23^ presented a small set with only 3(*m* + *n*) − 9 orthogonal product states and proved the local indistinguishability of these states in an *m* ⊗ *n* quantum system, where *m* ≥ 3 and *n* ≥ 3. Recently, Zhang *et al.*^24^ constructed 3*n* + *m* − 4 locally indistinguishable orthogonal product states that do not constitute a UPB and presented a smaller set with 2*n* − 1 orthogonal product states that cannot be perfectly distinguished by LOCC in *m* ⊗ *n* (3 ≤ *m* ≤ *n*). All the results show it is a meaningful work to research the structure of the locally indistinguishable product basis and the smallest number of locally indistinguishable orthogonal product states in arbitrary high-dimensional quantum systems.

In this paper, we construct a series of completable and locally indistinguishable orthogonal product bases, which have eight members, twelve members, ···, 4 min(*m*, *n*) − 4 members, respectively, in a general *m* ⊗ *n* (*m* ≥ 3 and *n* ≥ 3) quantum system. Our results show that Yu *et al.*’s conjecture^22^, i.e., any set of no more than 2(*d* − 1) product states is locally distinguishable in a *d* ⊗ *d* (*d* ≥ 3) quantum system, is not true. In fact, eight is so far the smallest number of locally indistinguishable states of a completable orthogonal product basis^22^,^23^,^24^. On the other hand, we construct a series of small and locally indistinguishable orthogonal product bases, which contain five members, seven members, ···, 2 min(*m*, *n*) − 1 members respectively, in *m* ⊗ *n* (*m* ≥ 3 and *n* ≥ 3). It should be pointed out that five is so far the smallest number of locally indistinguishable states of an orthogonal product basis by Refs. 20 and 21. These new results lead to a better understanding of the structures of locally indistinguishable product bases in arbitrary bipartite quantum systems.

## Results

Definition 1. *Consider a quantum system* 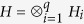 *with q parties. An orthogonal product basis (PB) is a set S of pure orthogonal product states spanning a subspace H*_*S*_ *of H. An uncompletable orthogonal product basis is a PB whose complementary subspace* 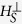
*, i.e., the subspace in H spanned by vectors that are orthogonal to all the vectors in H*_*S*_*, contains fewer mutually orthogonal product states than its dimension. An unextendible product basis (UPB) is an uncompletable product basis for which* 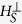 *contains no product state*^20^*. We call a PB is completable if it is not an uncompletable orthogonal product basis.*

Definition 2^20^. *Consider a multipartite quantum system* 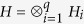 *with q parties. A strongly uncompletable product basis (SUCPB) is a PB spanning a subspace H*_*s*_ *in a locally extended Hilbert space H*_*ext*_ *such that for all H*_*ext*_ *the subspace* 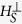 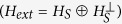 *contains fewer mutually orthogonal product states than its dimension.*

Definition 3. *Suppose that* {*M*_*t*_} *is a set of measurement operators, which can act on the measured Hilbert space. And t denote one of the possible measurement outcomes. If the measured state is* |*ϕ*〉 *before it is measured, the probability of the measurement outcome t is given by* 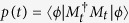 *and the postmeasurement state is* 
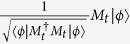
*. Furthermore, the measurement operators* {*M*_*t*_} *satisfy the completeness, i.e.,* 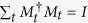
*. If we denote* 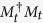 *as E*_*t*_, *it is easy to see that E*_*t*_ *is a positive semidefinite operator. We will say that the measurement is a positive operator-valued measure (POVM) and the objects E*_*t*_ *are the POVM elements corresponding to each measurement outcome t*^4^.

It is easy to see that POVM is a general measurement to a measured quantum state according to the definition 3. As we mentioned in the preceding part, LOCC denote local operations and classical communication. When it comes to identify a given state that is chosen from a known set of orthogonal states by LOCC, the local operations are local POVMs or local unitary operations. That is, if a measured state is a bipartite (or multipartite) quantum system, each party that holds one particle of the bipartite (or multipartite) quantum system can only perform POVM or unitary operations on his (or her) own particle. For simplicity, we usually say a set of orthogonal states is locally distinguishable if it can be distinguished by LOCC.

Definition 4^4^. *We will say that a POVM is trivial if all the POVM elements are proportional to the identity operator since such a measurement yields no information about the measured state. Any measurement not of this type will be called nontrivial.*

Different from Definition 4, we give a new definition about trivial measurement here. It should be noted that we say a measurement is trivial if it satisfies our new definition.

Definition 5. *A POVM is trivial to a set of orthogonal states,* 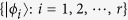
*, if and only if we cannot get any useful information about the measured state that is arbitrarily selected from the set by the POVM, i.e., for each of the POVM elements,* 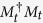
*, we have* 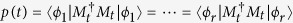.

Definition 6^4^. *Alice goes first if Alice is the first person to perform a nontrivial measurement upon the system.*

Lemma 1^20^. *Given a PB* 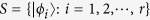 *on a Hilbert space* 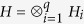 *of total dimension D. If the set S is completable in H or a locally extended Hilbert space H*_*ext*_, *then the density matrix* 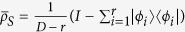 *is separable, where I is the identity matrix of rank D.*

### Local indistinguishability of completable orthogonal product basis

Now we construct a completable product basis with 4*p* − 4 members that cannot be locally distinguished in a general *m* ⊗ *n* (*m* ≥ 3 and *n* ≥ 3) quantum system and give a proof for its indistinguishability.

theorem 1. *In an m* ⊗ *n quantum system, the* 4*p* − 4 *orthogonal product states*


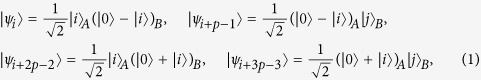


*cannot be perfectly distinguished by LOCC, where m* ≥ 3*, n* ≥ 3*, p is an arbitrary integer from* 3 *to* min(*m*, *n*)*, j* = *i* + 1 *when i* = 1, ···, *p* − 2 *and j* = 1 *while i* = *p* − 1.

*Proof*. Many proof techniques are borrowed from Ref. 22. To distinguish these states, one of the two parties (Alice and Bob) has to start with a nondisturbing measurement, i.e., the postmeasurement states should be mutually orthogonal. Without loss of generality, suppose that Alice goes first with a set of general *m* × *m* POVM elements 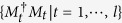, where


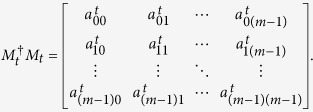


The post measurement states {*M*_*t*_ ⊗ *I*_*B*_|*ψ*_*i*_〉: *i* = 1, ···, 4*p* − 4} should be mutually orthogonal. For the states |*ψ*_*i*_〉 and |*ψ*_*j*_〉, where 1 ≤ *i* ≤ *p* − 1, 1 ≤ *j* ≤ *p* − 1 and *i* ≠ *j*, we have 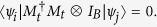 Thus 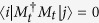, which means that 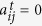 for 1 ≤ *i* ≤ *p* − 1, 1 ≤ *j* ≤ *p* − 1 and *i* ≠ *j*. For the states 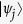 and 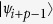, where *j* = *i* + 1 when *i* = 1, ···, *p* − 2 and *j* = 1 when *i* = *p* − 1, we can get 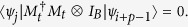 Thus 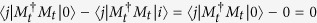, i.e., 
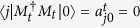
. Similarly, we can get 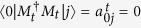. Therefore 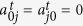 for *j* = 1, 2, ···, *p* − 1. For the states 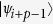 and 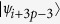, where *j* = *i* + 1 when *i* = 1, ···, *p* − 2 and *j* = 1 while *i* = *p* − 1, we have 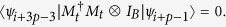 Thus 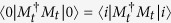. That is, 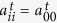 for *i* = 1, ···, *p* − 1.

Therefore, we have


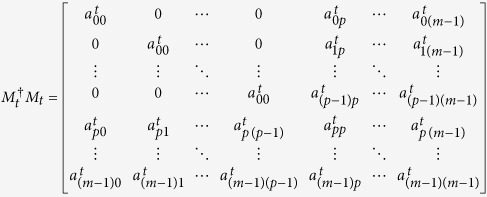


Now we consider the probability of the measurement outcome corresponding to the measurement operator *M*_*t*_ for each of the 4*p* − 4 states. It is easy to see that 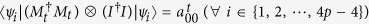 for *t* = 1, 2, ···, *l*, where 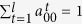 according to the completeness of the measurement operators. This means that any state of the 4*p* − 4 states can lead to the outcome that is corresponding to *M*_*t*_ with the same probability 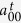, i.e., the measurement {*M*_*t*_ ⊗ *I*_*B*_ | *t* = 1, 2, ···, *l*} is trivial to the 4*p* − 4 states. In other words, Alice cannot get any information about which the measured state will be by the measurement {*M*_*t*_}. Thus Alice cannot go first. In fact, a similar argument can be used to exhibit that Bob faces the same dilemma, i.e., he cannot gain any useful information by a nondisturbing measurement, either. Therefore, they cannot perfectly distinguish these states by LOCC. In other words, the 4*p* − 4 states cannot be perfectly distinguished by LOCC. This completes the proof.

In general, the local indistinguishability of an incomplete PB are proved by Definition 4^4^,^22^,^23^. That is, in order to prove the local indistinguishability of an incomplete PB, we just need to show that all the POVM elements are proportional to the identity operator to keep the orthogonality of the postmeasurement states no matter who performs the first measurement. However, it is not a necessary condition for the local indistinguishability of an incomplete PB that all the POVM elements are proportional to the identity operator. In fact, we give a weaker condition to prove the local indistinguishability of an incomplete PB, which can be seen obviously by the proof of Theorem 1.

It is noted that the product basis (1) is completable since the 4*p* − 4 states of (1) can become a completed orthogonal product basis in *m* ⊗ *n* (*m* ≥ 3 and *n* ≥ 3) by adding the following *mn* − 4*p* + 4 states:


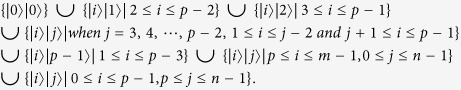


From Theorem 1, we know that the parameter *p* can be an arbitrary integer from 3 to min(*m*, *n*). That is, we actually construct a series of orthogonal product bases that are locally indistinguishable in *m* ⊗ *n* (*m* ≥ 3 and *n* ≥ 3). In particular, we have the following corollary by Theorem 1 when *p* = 3.

corollary 1. *In an m* ⊗ *n quantum system, the eight orthogonal product states*


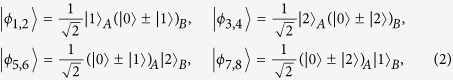


*cannot be perfectly distinguished by LOCC, where m* ≥ 3 *and n* ≥ 3.

As a special case, we can get eight states (2) that cannot be perfectly distinguished by LOCC in a *d* ⊗ *d* quantum system when *m* = *n* = *d* ≥ 3. This fact shows that the conjecture^22^, i.e., any set of no more than 2(*d* − 1) product states is locally distinguishable in a *d* ⊗ *d* (*d* ≥ 3) quantum system, is not true. By Refs 22, 23, 24, we know that the product basis composed by the eight states (2) is so far the smallest completable and locally indistinguishable orthogonal product basis in *m* ⊗ *n* (*m* ≥ 3 and *n* ≥ 3).

### Local indistinguishability of small orthogonal product basis

Now we give a small orthogonal product basis with 2*p* − 1 members that cannot be perfectly distinguished by LOCC in an *m* ⊗ *n* quantum system, where *m* ≥ 3, *n* ≥ 3 and 3 ≤ *p* ≤ min(*m*, *n*). Then we give a simple proof for its local indistinguishability.

theorem 2. *In an m* ⊗ *n quantum system, the* 2*p* − 1 *orthogonal product states*


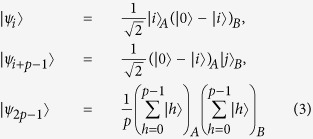


*cannot be perfectly distinguished by LOCC, where m* ≥ 3*, n* ≥ 3*, p is an arbitrary integer from* 3 *to min*(*m*, *n*)*, j* = *i* + 1 *when i* = 1, ···, *p* − 2 *and j* = 1 *while i* = *p* − 1.

*Proof.* Similar to the proof of Theorem 1, one of the two parties (Alice and Bob) has to start with a nondisturbing measurement to distinguish these states, i.e., the postmeasurement states should be mutually orthogonal. Without loss of generality, suppose that Alice goes first with a set of general *m* × *m* POVM elements 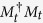 (*t* = 1, ···, *l*), where


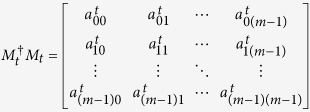


We can get 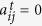 for 1 ≤ *i* ≤ *p* − 1, 1 ≤ *j* ≤ *p* − 1 and *i* ≠ *j*, and 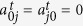 for *j* = 1, 2, ···, *p* − 1 by the same way as the proof of Theorem 1 since the postmeasurement states should be mutually orthogonal. For the states 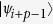 and |*ψ*_2*p*−1_〉, where *j* = *i* + 1 when *i* = 1, ···, *p* − 2 and *j* = 1 while *i* = *p* − 1, we have 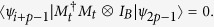 Thus 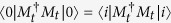, i.e., 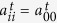 for *i* = 1, 2, ···, *p* − 1. Therefore, we have


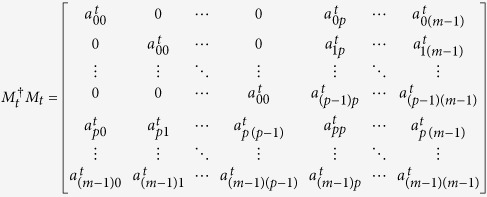


Now we consider the probability of the measurement outcome corresponding to the measurement operator *M*_*t*_ for each of the 2*p* − 1 states. It is easy to see that 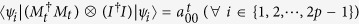 for *t* = 1, 2, ···, *l*, where 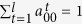 according to the completeness of the measurement operators. This means that any one of the 2*p* − 1 states can lead to the outcome that is corresponding to *M*_*t*_ with the same probability 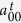, i.e., the measurement {*M*_*t*_ ⊗ *I*_*B*_ | *t* = 1, 2, ···, *l*} is trivial to the 2*p* − 1 states. That is, Alice cannot get any useful information about which the measured state will be by the measurement {*M*_*t*_}. In fact, if Bob goes first with a nondisturbing measurement, they cannot distinguish the 2*p* − 1 states, either. Therefore, the 2*p* − 1 states cannot be perfectly distinguished by LOCC. This completes the proof. □

The parameter *p* can be an arbitrary integer from 3 to min(*m*, *n*) in Theorem 2. That means that we construct a series of orthogonal product bases that are locally indistinguishable in *m* ⊗ *n* (*m* ≥ 3 and *n* ≥ 3). We have the following corollary directly by Theorem 2 when *p* = 3.

corollary 2. *In an m* ⊗ *n quantum system, the five orthogonal product states*


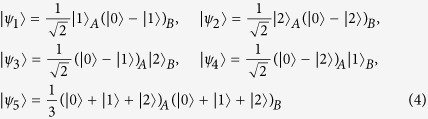


*are locally indistinguishable, where m* ≥ 3 *and n* ≥ 3.

When *m* = *n* = 3, the five states of (4) form a UPB. In Ref. 21, Bennett *et al.* exhibits two results. One is that a UPB is not completable even in a locally extended Hilbert space. The other is that if a set of orthogonal product states is exactly measurable by LOCC, then the set can be completed in some extended space. Thus it is obvious that the five states of (4) in *m* ⊗ *n* are locally indistinguishable by the two results, which is coincident with Corollary 2. Since any four orthogonal product states are shown to be locally distinguishable^20^, it is easy to see that five is the smallest number of uncompletable and locally indistinguishable product states.

Now we consider whether or not the 2*p* − 1 states of Theorem 2 are uncompletable in the *m* ⊗ *n* quantum system, where *m* ≥ 3, *n* ≥ 3 and *p* is an arbitrary integer from 3 to min(*m*, *n*). By the analysis of the last paragraph, we know that the 2*p* − 1 states of Theorem 2 are uncompletable in the *m* ⊗ *n* (*m* ≥ 3 and *n* ≥ 3) quantum system when *p* = 3. Then we prove that the 2*p* − 1 states of Theorem 2 are uncompletable in the *m* ⊗ *n* (*m* ≥ 4 and *n* ≥ 4) quantum system when *p* = 4. It is noted that some proof techniques are borrowed from Ref. 20. By Theorem 2, we get the following seven orthogonal product states that cannot be locally indistinguishable in the 4 ⊗ 4 quantum system.


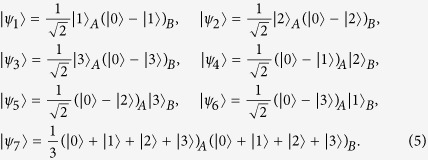


Let *S* = {|*ψ*_1_〉, |*ψ*_2_〉, ···, |*ψ*_7_〉}. The density matrix 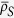 has rank 16 − 7 = 9. We can enumerate the product states that are orthogonal to the members of *S*, which are not all mutually orthogonal:


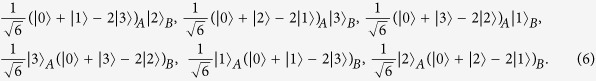


These six vectors are not enough to span the full Hilbert space 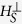. This means that the range of 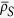 contains only six product states, whereas 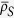 has rank 9. Therefore 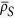 must be entangled. By Lemma 1, we can get *S* is a SUCPB because 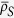 is entangled. That is, the 2*p* − 1 states of Theorem 2 are uncompletable in the *m* ⊗ *n* (*m* ≥ 4 and *n* ≥ 4) quantum system when *p* = 4. On the other hand, we can prove that the 2*p* − 1 states of Theorem 2 are uncompletable in the *m* ⊗ *n* (*m* ≥ 5 and *n* ≥ 5) quantum system by the same method, where 5 ≤ *p* ≤ min(*m*, *n*). By Theorem 2, we get the following 2*p* − 1 states in the *p* ⊗ *p* (*p* ≥ 5) quantum system.


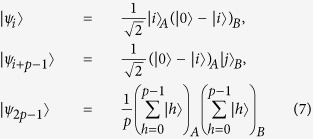


where *j* = *i* + 1 when *i* = 1, ···, *p* − 2 and *j* = 1 while *i* = *p* − 1. Let *S*′ = {|*ψ*_1_〉, |*ψ*_2_〉, ···, |*ψ*_2*p*−1_〉}. The density matrix 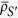 has rank *p*^2^ − (2*p* − 1) = (*p* − 1)^2^. We can enumerate the product states that are orthogonal to the members of *S*′, which are not all mutually orthogonal:


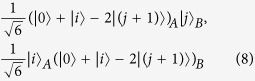


where *i* = 1, 2, ···, *p* − 1; *j* = *i* + 1 for *i* = 1, 2, ···, *p* − 2 while *j* = 1 for *i* = *p* − 1; and *j* + 1 = *i* + 2 for *i* = 1, 2, ···, *p* − 3 while *j* + 1 = 1 for *i* = *p* − 2 and *j* + 1 = 2 for *i* = *p* − 1. These 2*p* − 2 vectors are not enough to span the full Hilbert space 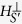. This means that the range of 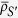 contains only 2*p* − 2 product states, whereas 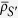 has rank (*p* − 1)^2^. Therefore 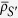 must be entangled. By Lemma 1, we can get *S*′ is a SUCPB because 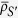 is entangled. That is, the 2*p* − 1 states of Theorem 2 are uncompletable in the *m* ⊗ *n* (*m* ≥ 5 and *n* ≥ 5) quantum system when 5 ≤ *p* ≤ min(*m*, *n*). Therefore, the 2*p* − 1 orthogonal product states of (3) are uncompletable in the *m* ⊗ *n* quantum system, where *m* ≥ 3, *n* ≥ 3 and *p* is an arbitrary integer from 3 to min(*m*, *n*).

## Discussion

In this paper, we construct a completable orthogonal product basis with 4*p* − 4 members that cannot be perfectly distinguished by LOCC in an *m* ⊗ *n* quantum system, where *m* ≥ 3, *n* ≥ 3 and *p* is an arbitrary integer from 3 to min(*m*, *n*), and give a simple but quite effective proof. As a special case, we get eight orthogonal product states that can be completable but cannot be locally distinguished in *m* ⊗ *n* (*m* ≥ 3 and *n* ≥ 3). On the other hand, we construct a samll locally indistinguishable orthogonal product basis with 2*p* − 1 members in *m* ⊗ *n*, which are uncompletable, where *m* ≥ 3, *n* ≥ 3 and *p* is an arbitrary integer from 3 to min(*m*, *n*). Our work is useful to understand the structures both of completable and uncompletable product bases that cannot be distinguished by LOCC in arbitrary bipartite quantum system.

## Additional Information

**How to cite this article**: Xu, G.-B. *et al.* Locally indistinguishable orthogonal product bases in arbitrary bipartite quantum system. *Sci. Rep.* **6**, 31048; doi: 10.1038/srep31048 (2016).

## References

[b1] Bennett C. H. Quantum cryptography using any two nonorthogonal states. Phys. Rev. Lett. 68, 3121 (1992). 10045619 10.1103/PhysRevLett.68.3121

[b2] Bergou J. A., Herzog U. & Hillery M. Quantum filtering and discrimination between sets of boolean functions. Phys. Rev. Lett. 90, 257901 (2003). 12857168 10.1103/PhysRevLett.90.257901

[b3] Bennett C. H. *et al.* Quantum nonlocality without entanglement. Phys. Rev. A 59, 1070 (1999).

[b4] Walgate J. & Hardy L. Nonlocality, asymmetry, and distinguishing bipartite states. Phys. Rev. Lett. 89, 147901 (2002). 12366075 10.1103/PhysRevLett.89.147901

[b5] Walgate J., Short A. J., Hardy L. & Vedral V. Local distinguishability of multipartite orthogonal quantum states. Phys. Rev. Lett. 85, 4972 (2000). 11102164 10.1103/PhysRevLett.85.4972

[b6] Chen J. X. & Johnston N. The minimum size of unextendible product bases in the bipartite case (and some multipartite cases). Commun. Math. Phys. 333, 351–365 (2015).

[b7] Bravyi S. B. Unextendible product bases and locally unconvertible bound entangled states. Quantum Inf. Process. 3, 309–329 (2004).

[b8] Childs A. M. *et al.* A framework for bounding nonlocality of state discrimination. Commun. Math. Phys. 323, 1121–1153 (2013).

[b9] Chen P. X. & Li C. Z. Orthogonality and distinguishability: Criterion for local distinguishability of arbitrary orthogonal states. Phys. Rev. A 68, 062107 (2003).

[b10] Niset J. & Cerf N. J. Multipartite nonlocality without entanglement in many dimensions. Phys. Rev. A 74, 052103 (2006).

[b11] Jiang W., Ren X. J., Wu Y. C., Zhou Z. W, Guo G. C. & Fan H. A sufficient and necessary condition for 2*n* − 1 orthogonal states to be locally distinguishable in a *C*^2^ ⊗ *C*^*n*^ system. J. Phys. A: Math. Theor. 43, 325303 (2010).

[b12] Yu N. K., Duan R. Y. & Ying M. S. Any 2 ⊗ *n* subspace is locally distinguishable. Phys. Rev. A 84, 012304 (2011).

[b13] Xin Y. & Duan R. Y. Local distinguishability of orthogonal 2 ⊗ 3 pure states. Phys. Rev. A 77, 012315 (2008).

[b14] Yang Y. H., Gao F., Tian G. J., Cao T. Q. & Wen Q. Y. Local distinguishability of orthogonal quantum states in a 2 ⊗ 2 ⊗ 2 system. Phys. Rev. A 88, 024301 (2013).

[b15] Duan R. Y., Xin Y. & Ying M. S. Locally indistinguishable subspaces spanned by three-qubit unextendible product bases. Phys. Rev. A 81, 032329 (2010).

[b16] Chen P. X. & Li C. Z. Distinguishing the elements of a full product basis set needs only projective measurements and classical communication. Phys. Rev. A 70, 022306 (2004).

[b17] Rinaldis S. D. Distinguishability of complete and unextendible product bases. Phys. Rev. A 70, 022309 (2004).

[b18] Ma T., Zhao M. J., Wang Y. K. & Fei S. M. Noncommutativity and local indistinguishability of quantum states. Sci. Rep. 4, 6336 (2014). 25208830 10.1038/srep06336 PMC4160716

[b19] Feng Y. & Shi Y. Y. Characterizing locally indistinguishable orthogonal product states. IEEE Trans. Inf. Theory 55, 2799 (2009).

[b20] DiVincenzo D. P., Mor T., Shor P. W., Smolin J. A. & Terhal M. Unextendible product bases, uncompleteable product bases and bound entanglement. Commun. Math. Phys. 238, 379 (2003).

[b21] Bennett C. H. *et al.* Unextendible product bases and bound entanglement. Phys. Rev. Lett. 82, 5385 (1999).

[b22] Yu S. X. & Oh C. H. Detecting the local indistinguishability of maximally entangled states. arXiv: 1502.01274v1[quant-ph] (2015).

[b23] Wang Y. L., Li M. S., Zheng Z. J. & Fei S. M. Nonlocality of orthogonal product-basis quantum states. Phys. Rev. A 92, 032313 (2015).

[b24] Zhang Z. C., Gao F., Cao Y., Qin S. J & Wen Q. Y. Local indistinguishability of orthogonal product states. Phys. Rev. A 93, 012314 (2016).

